# DNA Oxidative Damage as a Sensitive Genetic Endpoint to Detect the Genotoxicity Induced by Titanium Dioxide Nanoparticles

**DOI:** 10.3390/nano12152616

**Published:** 2022-07-29

**Authors:** Zhangjian Chen, Jiaqi Shi, Yi Zhang, Shuo Han, Jiahe Zhang, Guang Jia

**Affiliations:** 1 Department of Occupational and Environmental Health Sciences, School of Public Health, Peking University, Beijing 100191, China; zhangjianchen@pku.edu.cn (Z.C.); 1610306221@pku.edu.cn (J.S.); 1710306142@pku.edu.cn (Y.Z.); hansure@pku.edu.cn (S.H.); zhangjh_pku15@126.com (J.Z.); 2 Beijing Key Laboratory of Toxicological Research and Risk Assessment for Food Safety, Peking University, Beijing 100191, China

**Keywords:** genotoxicity, titanium dioxide nanoparticles, DNA oxidative damage, Fpg-modified comet assay, oxidative stress

## Abstract

The genotoxicity of nanomaterials has attracted great attention in recent years. As a possible occupational carcinogen, the genotoxic effects and underlying mechanisms of titanium dioxide nanoparticles (TiO_2_ NPs) have been of particular concern. In this study, the effect of TiO_2_ NPs (0, 25, 50 and 100 µg/mL) on DNA damage and the role of oxidative stress were investigated using human bronchial epithelial cells (BEAS-2B) as an in vitro model. After detailed characterization, the cytotoxicity of TiO_2_ NPs was detected. Through transmission electron microscopy (TEM), we found that TiO_2_ NPs entered the cytoplasm but did not penetrate deep into the nucleus of cells. The intracellular levels of reactive oxygen species (ROS) significantly increased in a dose-dependent manner and the ratios of GSH/GSSG also significantly decreased. The results of the normal comet assay were negative, while the Fpg-modified comet assay that specifically detected DNA oxidative damage was positive. Meanwhile, *N*-acetyl-L-cysteine (NAC) intervention inhibited the oxidative stress and genotoxicity induced by TiO_2_ NPs. Therefore, it was suggested that TiO_2_ NPs could induce cytotoxicity, oxidative stress and DNA oxidative damage in BEAS-2B cells. DNA oxidative damage may be a more sensitive genetic endpoint to detect the genotoxicity of TiO_2_ NPs.

## 1. Introduction

The size effect and high surface activity of nanoparticles make it easy to penetrate the cell membrane to directly affect the genetic material of cells, or indirectly induce chromosome or DNA breakage through mechanisms, such as oxidative stress [1,2,3,4]. Research on the genotoxicity and potential carcinogenicity of nanomaterials plays an important role in their safety evaluation [5,6]. However, there are certain peculiarities in the interaction of nanomaterials with organisms, which are different from most chemicals and environmental mutagens, so that the current routinely used genotoxicity standardized methods may not be effective and reliable for nanomaterials. Both the Food and Drug Administration (FDA) in the USA and the Organization for Economic Co-operation and Development (OECD) expressed the need to evaluate whether traditional standard genotoxicity methods are applicable to nanomaterials [7]. However, previous studies still mostly used traditional methods to evaluate the genotoxicity of nanomaterials, which may be one of the main reasons for the conflicting research conclusions. Landsiedel et al. [8] believed that the Ames test was not suitable for the evaluation of genotoxicity of nanomaterials due to the difficulty in nanomaterials passing through the cell wall of bacteria and this conclusion was also supported by subsequent studies [9]. Therefore, exploring nanomaterial-sensitive endpoints or methods of genotoxicity is of great significance for the safety evaluation of nanomaterials.

Titanium dioxide nanoparticles (TiO_2_ NPs) have become one of the most widely used nanomaterials due to their peculiar color effect and superior ultraviolet absorption ability. TiO_2_ is a chemical with high output and traditional TiO_2_ coarse particles are considered to be representative of poorly soluble, low-toxicity (PSLT) particles. However, many recent toxicological studies have shown that the toxicity of TiO_2_ NPs is significantly higher than that of TiO_2_ coarse particles [10,11,12,13]. Robichaud et al. estimated that the production of nanoscale TiO_2_ in the United States exceeded 260,000 tons in 2015, accounting for approximately 10% of the total TiO_2_ market, and this proportion is expected to be as high as 100% by 2025 [14]. As the production of TiO_2_ NPs continues to increase, the occupational population exposed to TiO_2_ NPs in the process of production, transportation, storage and use will also gradually increase. Meanwhile, in the environmental life cycle of nanomaterials, occupational exposure is primary in all exposure situations. Therefore, the occupational health risks of inhaling TiO_2_ NPs through the respiratory tract need urgent attention. The National Institute for Occupational Safety and Health (NIOSH) recommended an occupational exposure limit (OEL) for TiO_2_ NPs in workplace air, which is only 1/8 of that for TiO_2_ fine particles (>100 nm). This is mainly based on a large number of in vivo experiments showing that TiO_2_ NPs can be deposited in various parts of the respiratory tract after inhalation, causing lung inflammation, lung injury, fibrosis and even tumors [15,16]. Both NIOSH and the International Agency for Research on Cancer (IARC) consider TiO_2_ NPs a potential human carcinogen [17]. Therefore, the research and evaluation of genotoxicity for TiO_2_ NPs have received much attention, which is of great significance for predicting the carcinogenicity and clarifying the relevant mechanisms.

In fact, research on the genotoxicity of TiO_2_ NPs has continued to emerge in the past decade, but the results are still so conflicting that no clear conclusions can be drawn. This is probably because the genotoxicity of TiO_2_ NPs is affected by many factors, such as their physicochemical properties and experimental methodological differences, including different genetic endpoint detection methods and whether to consider the effect of light, etc. [18,19,20]. Our previous study showed that TiO_2_ NPs could induce HPRT gene mutation in V79 cells and DNA double-strand breaks in rat bone marrow cells after oral administration, but no obvious chromosomal damage was found in the micronuclei assay [21]. Subsequently, some other studies also reported that TiO_2_ NPs could cause genotoxicity, suggesting that TiO_2_ NPs are likely to be genotoxic to humans [2,22,23,24,25]. However, one-by-one evaluation and research may be required for TiO_2_ NPs with different properties and in different exposure scenarios. Meanwhile, more sensitive or appropriate methods also need to be evaluated in the genotoxicity study of nanomaterials.

This study aimed to investigate the genotoxic effects and underlying mechanisms of TiO_2_ NPs under occupational respiratory exposure. The effect of TiO_2_ NPs on DNA damage and the role of oxidative stress were investigated using human bronchial epithelial cells (BEAS-2B) as an in vitro model. In addition to the normal comet assay, the Fpg-modified comet assay that specifically detects DNA oxidative damage was also carried out simultaneously. The design of the present study took full account of the consensus proposed in the OECD report on the assessment of the genotoxicity of nanomaterials [26].

## 2. Materials and Methods

### 2.1. Characterization of Physicochemical Properties of TiO_2_ NPs

TiO_2_ NPs were purchased from Shanghai Macklin Biochemical Co., Ltd. (Shanghai, China). The detailed characterization methods and physicochemical properties of TiO_2_ NPs were described in our published paper [27]. TiO_2_ NPs were characterized in terms of particle size, purity, crystal form, hydrodynamic diameters and zeta potential. Transmission electron microscopy (TEM, JEM-1400, JEOL Company, Tokyo, Japan) was used to observe the particle morphology and measure the particle size distribution by image analysis. An X-ray energy spectrum analyzer (EDS, Nova_NanoSEM430, FEI Company, Hillsboro, OR, USA) was used to analyze the composition and content of elements of nanoparticles. X-ray powder diffractometry (XRD, PANalytical’s X’Pert PRO, X’Celerator, EA Almelo, The Netherlands) was used to determine the crystal form. The specific surface area was measured by Brunauer–Emmett–Teller method. The hydrodynamic diameters and zeta potentials of TiO_2_ NPs (100 µg/mL) in ultrapure water and Dulbecco’s modified Eagle medium (DMEM) were measured by ZetaSizer Nano ZS90 (Malvern Instruments Ltd., Malvern, UK). Considering the photocatalytic activity of TiO_2_ NPs and its influence on biological effects including genotoxicity [19,20], during the storage and use of TiO_2_ NPs, the light-shielding operation is performed as much as possible.

### 2.2. Cell Culture and Exposure to TiO_2_ NPs

Human normal bronchial epithelial cells (BEAS-2B) were obtained from the American Type Culture Collection (ATCC). BEAS-2B cells were cultured in Dulbecco’s Modified Eagle Medium (DMEM, Gibco) supplemented with 10% fetal bovine serum (FBS, HyClone, Thermo Fisher Scientific, Waltham, MA, USA) and 4 mM glutamine at 37 °C in a humidified atmosphere containing 5% CO_2_. When the cell confluence reached 90%, the cells were passaged by 0.25% trypsin with EDTA every 2 days and the passage ratio was 1:2.

Logarithmic growth phase BEAS-2B cells were exposed to the suspensions of TiO_2_ NPs (0, 25, 50, 100 µg/mL) in serum-free DMEM for 48 h, sonicated for 15 min and freshly prepared before each exposure. Three biological replicates were performed in each group.

### 2.3. Cytotoxicity Assay

The cytotoxicity of TiO_2_ NPs at different concentrations on BEAS-2B cells was evaluated by Cell Counting Kit-8 (CCK-8) assay. The basic principle of the kit is that the amount of formazan produced is directly proportional to the number of living cells. BEAS-2B cells in 96-well plates were exposed to 0–200 µg/mL TiO_2_ NPs for 24 h and 48 h. Then, 10 µL of CCK-8 solution was added to each well and incubated for 2 h. The absorbance of formazan was measured at 450 nm for each well by a microplate reader, using the absorbance at 600 nm as a reference calibration for cancellation of the signal alteration of turbidity in the solution caused by NPs. According to the results of the cytotoxicity experiment, the exposure dose and time for follow-up experiments were selected with a significant decrease in cell activity but still greater than 70%.

### 2.4. Detection of Oxidative Stress Biomarkers

BEAS-2B cells were seeded into 6-well plates and exposed to TiO_2_ NPs at 0, 25, 50 and 100 µg/mL for 48 h. The level of intracellular reactive oxygen species (ROS) was detected by 2′,7′-Dichlorofluorescin diacetate (DCFH-DA) probe, which can be oxidized by intracellular ROS to fluorescent DCF. The fluorescence intensity is proportional to the amount of ROS and was detected by a flow cytometer. Positive controls were processed with 98 mM hydrogen peroxide for 5 min at room temperature. The levels of reduced glutathione (GSH) and oxidized glutathione glutathione (GSSG) were detected by glutathione test kit (Nanjing Jiancheng, China). The kit utilizes the chromogenic reaction in which the substrate 5,5′-dithiobis-2-nitrobenoic acid (DTNB) can be reduced to yellow TNB. During the detection process, the reagents were prepared in strict accordance with the kit instructions. For formal detection, a 96-well plate was used and 10 µL of sample and 150 µL of total glutathione detection working solution were added to each well. After 5 min of incubation at room temperature, 50 µL of 0.5 mg/mL NADPH solution was added to each well, mixed and incubated at room temperature for 25 min. Immediately afterwards, the absorbance at 405 nm was measured with a microplate reader.

### 2.5. Comet Assay

The comet assay, also known as single-cell gel electrophoresis, can move the broken DNA fragments to the anode by electrophoresis. After staining, a comet-like pattern can be observed under a fluorescence microscope. It is a traditional method for detecting DNA single- and double-strand breaks. In this study, the normal comet assay and the formamidopyrimidine DNA glycosyla (Fpg) modified comet assay were used to detect the genotoxicity of TiO_2_ NPs. The cells were seeded into 6-well plates and exposed to TiO_2_ NPs at 0, 25, 50 and 100 µg/mL for 48 h or positive control of hydrogen peroxide at 1.53 mM for 5 min at room temperature. After exposure, the cells were collected by trypsinization and centrifugation, resuspended in PBS and stored at 4 °C until gel coating. Gel coating was prepared by mixing 60 µL of cell suspension with 60 µL of 1.0% low-melting-point agarose on slides precoated with 180 µL of 0.5% agarose. Then, the slides were lysed at 4 °C for 1 h in precooled lysis buffer and transferred into the electrophoresis tank for alkaline unwinding for 20 min. After the unwinding was completed in the dark, electrophoresis was started at 4 °C. The voltage was maintained at 25 V and the current was 300 mA for 20 min. After electrophoresis, the slides were soaked in the neutralizing solution for 30 min, and a new neutralizing solution replaced the previous during the process. Then, the slides were taken out and 3~4 drops of GelredTM working solution were added in the dark room and detected at an excitation wavelength of 302/312 nm by a fluorescence microscope as soon as possible. At least 10 images and 100 cells for each sample were randomly selected to perform statistics on Olive tail moment (OTM, a product of the median migration distance and the percentage of DNA in the tail) and comet tail DNA percentage (% tail DNA) using CaspLab software.

The steps in the Fpg-modified comet assay were basically the same as those of the normal comet assay, but an additional enzymatic digestion step was added. After the lysis, the slides were placed in enzyme buffer and washed 3 times for 5 min each time. Enzyme buffer was used to dilute 8000 U/mL Fpg at 1:3000 into the working solution, 50 µL of the enzyme working solution was added to each sample. The slides were placed in a wet box at 37 °C and incubated for 45 min. After the incubation, the subsequent unwinding and electrophoresis steps were performed.

### 2.6. Detection of γ-H_2_AX by Indirect Immunofluorescence

The phosphorylation of histone H_2_AX on serine 139 (γ-H_2_AX), a marker of DNA double-strand breaks, was evaluated by indirect immunofluorescence staining. Indirect immunofluorescence is a fluorescence imaging technique coupling fluorescein to a specific target antigen through an antibody, and the combination of multiple secondary antibodies and primary antibodies can amplify the fluorescent signal and increase the sensitivity of detection. The logarithmic growth cells were taken and seeded in confocal small dishes. After the cells adhered, the cells were exposed to TiO_2_ NPs at 0, 25, 50 and 100 µg/mL for 48 h. Three biological replicates were set in each group. The cells were fixed with 4% paraformaldehyde for 15 min and treated with 1% Triton X-100 prepared in PBS for 15 min at room temperature. Then, blocking solution containing 10% goat serum was added and the cells were blocked for 30 min at 37 °C. Then, γ-H_2_AX rabbit monoclonal antibody diluted 1:500 in blocking solution was directly added and incubated at 4 °C overnight. After incubation, the goat anti-rabbit antibody (AlexaFluor488-labeled green fluorescent antibody) diluted 1:200 in blocking solution was incubated for 1 h at room temperature in the dark and washed three times with PBS for 5 min subsequently. We then added 3–4 drops of fluorescent mounting medium containing DAPI to the middle glass slide in each dish. At least 100 cells were counted in each sample by a confocal microscope and the positive cell rate was calculated.

### 2.7. NAC Antioxidant Intervention Experiment

*N*-acetyl-L-cysteine (NAC), with antioxidative and free radical scavenging effects, is a commonly used antioxidant. This study adopted the NAC antioxidant intervention design to explore the role of oxidative stress in the genotoxicity of TiO_2_ NPs. For the intervention design, four experimental groups were set up, including the control group, NAC (5 mM) group, TiO_2_ NPs (100 µg/mL) group and NAC (5 mM) + TiO_2_ NPs (100 µg/mL) group with exposure time of 48 h. The levels of ROS and GSH/GSSG in cells after NAC intervention were detected to verify the intervention effect, and the changes in genotoxicity after NAC intervention were detected by comet assay and indirect immunofluorescence, of which the methods were consistent with the above.

### 2.8. Statistical Analysis

R 3.6.3 was used for statistical analysis of experimental data. The Shapiro–Wilk normality test was used to test whether the experimental data conformed to normal distribution and Bartlett’s method was used to test whether the data conformed to the homogeneity of variance. For continuous variables conforming to normal distribution, data were expressed as mean ± standard deviation (S.D.), otherwise expressed as the median and interquartile range (IQR). For data with normality and homogeneity of variance, one-way analysis of variance (ANOVA) was used, Dunnett-t test was used for comparisons between the treatment group and the control group and the LSD method was used for pairwise comparisons between each group. For nonnormal or unequal variance data, the Kruskal–Wallis rank-sum test was used and the Nemenyi test was used for pairwise comparisons between groups. All tests were two-sided and *p* < 0.05 was considered to be statistically significant.

## 3. Results

### 3.1. Characterization of the TiO_2_ NPs

**Figure 1:**
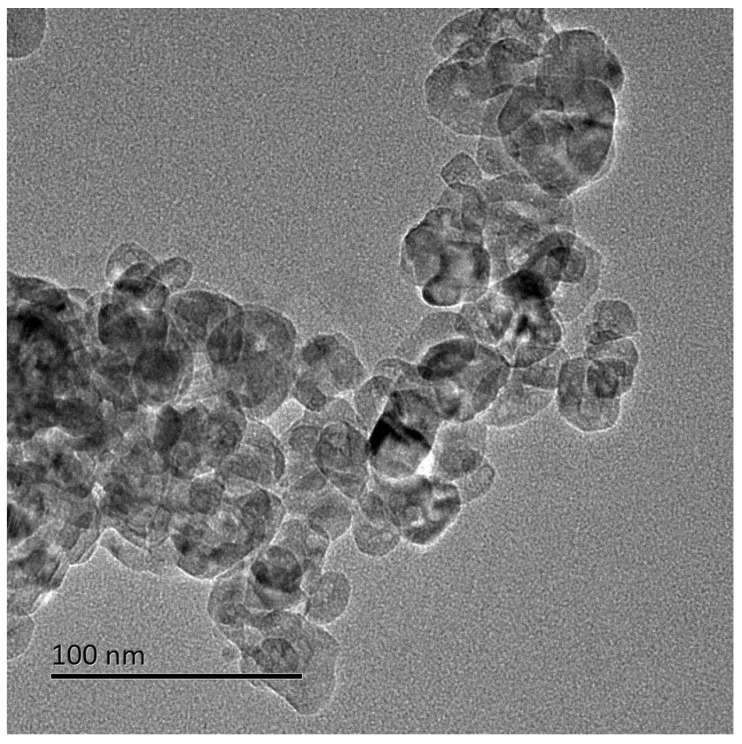
Transmission electron microscopy (TEM) image of TiO_2_ NPs.

The TiO_2_ NPs used in this study were spherical, with primary particle sizes of 25.12 ± 5.64 nm (Figure 1). The crystal form was anatase with a BET specific surface area of 77.51 ± 0.29 m^2^/g. The hydrodynamic diameter and zeta potential of TiO_2_ NPs (100 µg/mL) in ultrapure water were 609.43 ± 60.35 nm and −8.33 ± 0.22 mV, respectively, but in DMEM for cell culture were 878.93 ± 105.75 nm and −15.20 ± 0.92 mV, respectively. The aggregation of TiO_2_ NPs occurred in solutions, which was greater in DMEM than in ultrapure water.

### 3.2. TiO_2_ NPs Induced Cytotoxicity in BEAS-2B Cells

**Figure 2:**
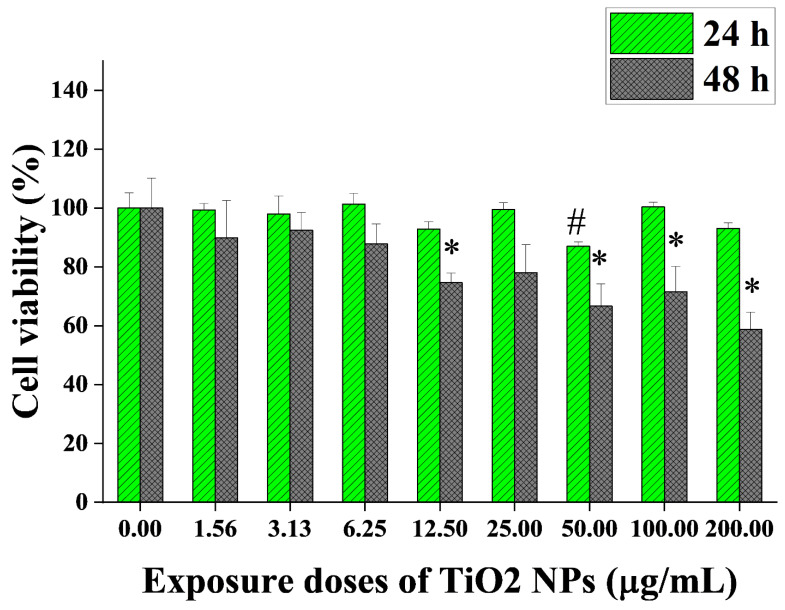
Cytotoxicity induced by TiO_2_ NPs in BEAS-2B cells (mean ± SD, n = 3). BEAS-2B cells were exposed to 0–200 µg/mL TiO_2_ NPs for 24 h and 48 h. Significant difference from the control group for 24 h exposure (# *p* < 0.05); significant difference from the control group for 48 h exposure (* *p* < 0.05).

After exposure to 0–200 µg/mL TiO_2_ NPs for 24 h and 48 h, the cytotoxicity induced by TiO_2_ NPs in BEAS-2B cells was measured by the CCK-8 method. The results are shown in Figure 2. The BEAS-2B cell viability was significantly decreased in TiO_2_ NP-exposed groups at 24 and 48 h. After 24 h of exposure, the cell viability of the 50 µg/mL group was 87%, which was significantly lower than that of the control group, but in the subsequent two higher-dose groups (100 and 200 µg/mL), the cell viability recovered to no difference from the control group. After 48 h of exposure, the dose–response relationship was more obvious. The cell viability of the 12.50, 50, 100 and 200 µg/mL groups at 48 h was significantly lower than that of the control group, which was reduced to 74.6%, 66.6%, 71.5% and 58.7%, respectively. As significant cytotoxicity would easily cause apoptosis, which may be unfavorable to the detection of genotoxicity, we chose 100 µg/mL as the maximum dose.

### 3.3. Cellular Uptake of TiO_2_ NPs Observed by TEM

**Figure 3:**
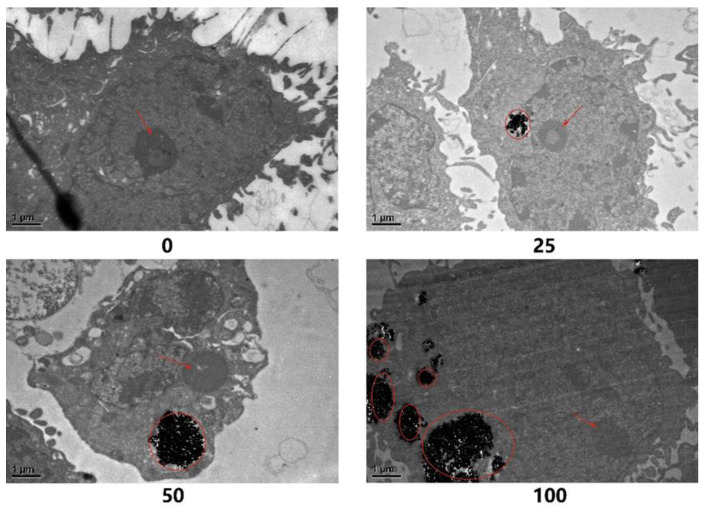
TEM observation of the cellular uptake of TiO_2_ NPs in BEAS-2B cells after exposure for 48 h. The magnification was 15,000×. The nanoparticles were circled by red ovals and the red arrows indicated the nucleoli of the nucleus. The numbers marked below the pictures are the exposure doses in µg/mL.

The extent of cellular uptake is a critical factor to consider when interpreting the genotoxicity results of nanomaterials. As shown in Figure 3, the cell morphology and nanoparticle distribution in BEAS-2B cells after exposure to TiO_2_ NPs for 48 h were observed. TiO_2_ NPs could be seen to enter the cytoplasm but not into the nucleus, suggesting that a direct interaction between TiO_2_ NPs and DNA in the nucleus may not occur. In addition, the number of nanoparticles entering the cell increased with the dose. Meanwhile, obvious agglomeration of TiO_2_ NPs in the cells was also observed in the exposure groups, among which the most serious agglomeration was found in the 100 µg/mL group. The location of TiO_2_ NPs in cells suggested that they may induce genotoxicity through an indirect pathway, even though the direct pathway could not be completely ruled out.

### 3.4. DNA Damage Induced by TiO_2_ NPs

**Figure 4:**
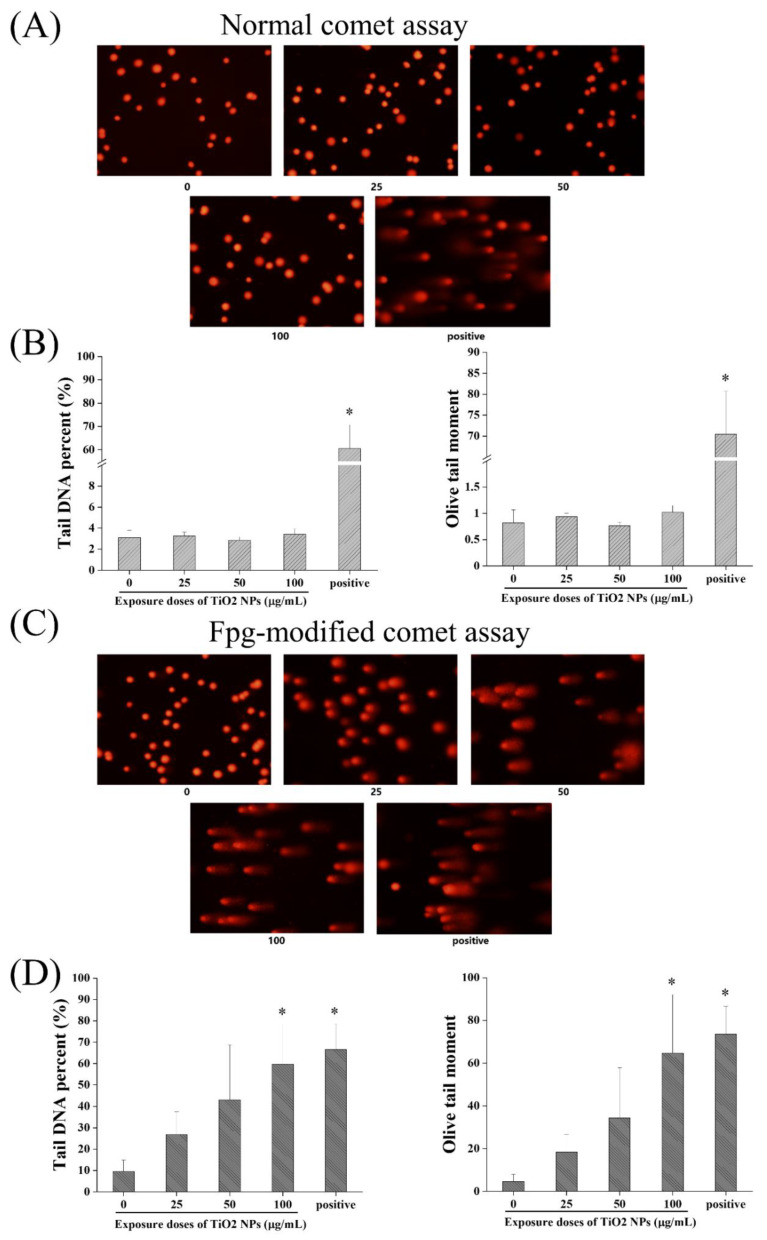
Effects of TiO_2_ NPs on DNA damage in BEAS-2B cells using the comet assay. BEAS-2B cells were exposed to TiO_2_ NPs at doses of 25, 50 and 100 µg/mL for 48 h or the positive control of hydrogen peroxide (H_2_O_2_) at 1.53 mM for 5 min. (**A**) In the normal comet assay, no obvious comet cells were found, except for the positive control group. (**B**) Quantitative analysis showed no significant difference between different groups. (**C**) In the Fpg-modified comet assay, comet cells were found in the TiO_2_ NP exposure groups. (**D**) Quantitative analysis of the Fpg-modified comet assay showed that both the Olive tail moment and comet tail DNA percentage in the 100 µg/mL TiO_2_ NP group were significantly higher than those in the control group. Significant difference from the control (* *p* < 0.05).

DNA damage in BEAS-2B cells was evaluated by comet assay and immunofluorescence detection of γ-H_2_AX after exposure to TiO_2_ NPs at doses of 25, 50 and 100 µg/mL for 48 h. In addition to the normal comet assay, the Fpg-modified comet assay that specifically detected DNA oxidative damage was also carried out simultaneously. As shown in Figure 4, the positive control had a significant comet-like electrophoresis pattern, which verified the accuracy of the experimental method. In the normal comet assay, no obvious difference was observed between different groups. However, in the Fpg-modified comet assay, both the Olive tail moment and comet tail DNA percentage in the 100 µg/mL TiO_2_ NP group were significantly higher than those in the control group. Therefore, TiO_2_ NPs mainly caused DNA oxidative damage in BEAS-2B cells.

**Figure 5:**
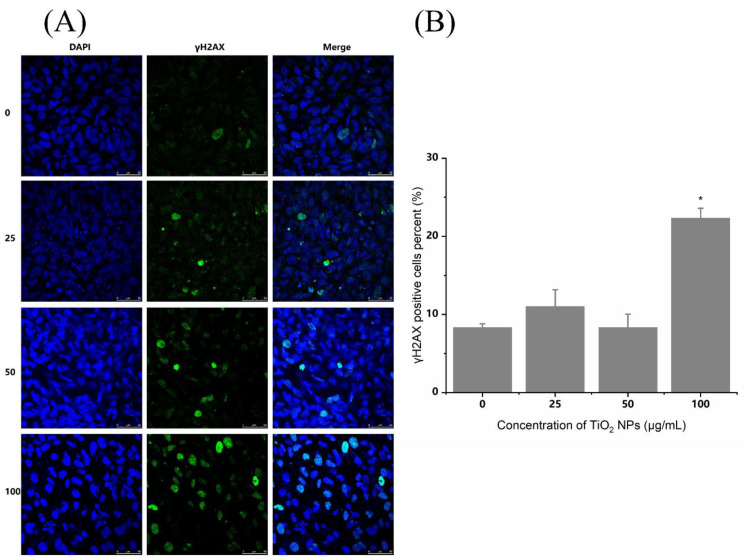
Effects of TiO_2_ NPs on intracellular γ-H_2_AX in BEAS-2B cells. As a marker of DNA double-strand breaks (DSBs), γ-H_2_AX was detected by indirect immunofluorescence staining after exposure to TiO_2_ NPs at doses of 25, 50 and 100 µg/mL for 48 h. (**A**) The γ-H_2_AX stained with green fluorescence was mainly distributed in the nucleus stained with blue DAPI fluorescence. (**B**) The percent of γ-H_2_AX positive cells increased significantly in the 100 µg/mL TiO_2_ NP group, compared with the control group. Significant difference from the control (* *p* < 0.05).

γ-H_2_AX is a marker of DNA double-strand breaks (DSBs). As shown in Figure 5, the percentage of γ-H_2_AX-positive cells increased significantly in the 100 µg/mL TiO_2_ NP group, compared with the control group. However, the results did not show a dose–response relationship. Meanwhile, γ-H_2_AX stained with green fluorescence was mainly distributed in the nucleus stained with blue DAPI fluorescence. Therefore, these results showed that TiO_2_ NPs could also induce a certain degree of DNA double-strand breaks in BEAS-2B cells, even if the normal comet assay could not detect it.

### 3.5. The Role of Oxidative Stress in DNA Damage Induced by TiO_2_ NPs

**Figure 6:**
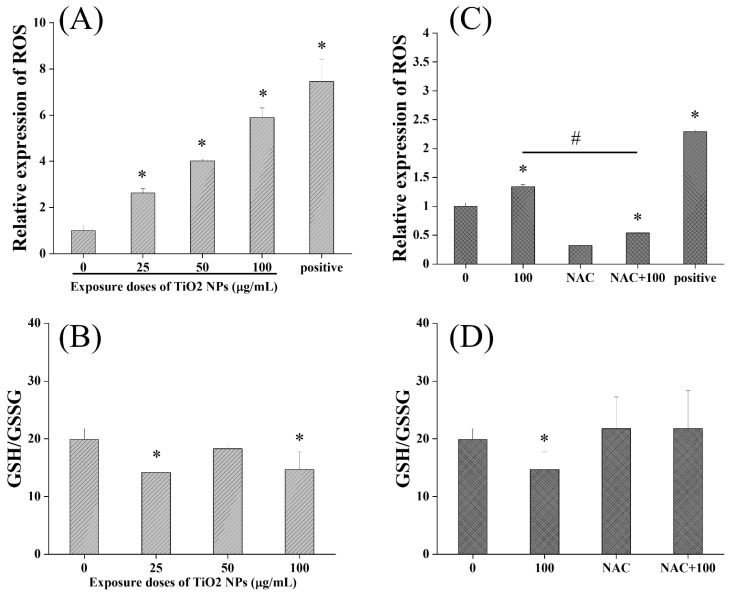
Effects of TiO_2_ NPs on oxidative stress in BEAS-2B cells. (**A**) TiO_2_ NPs induced significantly increased levels of ROS in BEAS-2B cells with a good dose–response relationship. (**B**) TiO_2_ NPs induced a significant decrease in GSH/GSSG at doses of 25 and 100 µg/mL. (**C**,**D**) After antioxidant intervention by NAC, the oxidative stress induced by TiO_2_ NPs was significantly recovered. Significant difference from the control (* *p* < 0.05), significant difference from the 100 µg/mL TiO_2_ NP group (# *p* < 0.05).

As shown in Figure 6, TiO_2_ NPs induced significantly increased levels of ROS in BEAS-2B cells, with a good dose–response relationship. The level of cellular ROS in the 100 µg/mL group increased by 4.9-times compared with the control group, indicating that the production of ROS was significantly activated by TiO_2_ NPs. Meanwhile, compared with the control group, the cellular GSH/GSSG ratios in the 25 and 100 µg/mL groups were significantly decreased, indicating that cellular antioxidant capacity was weakened. Taken together, TiO_2_ NPs induced obvious oxidative stress in BEAS-2B cells.

**Figure 7:**
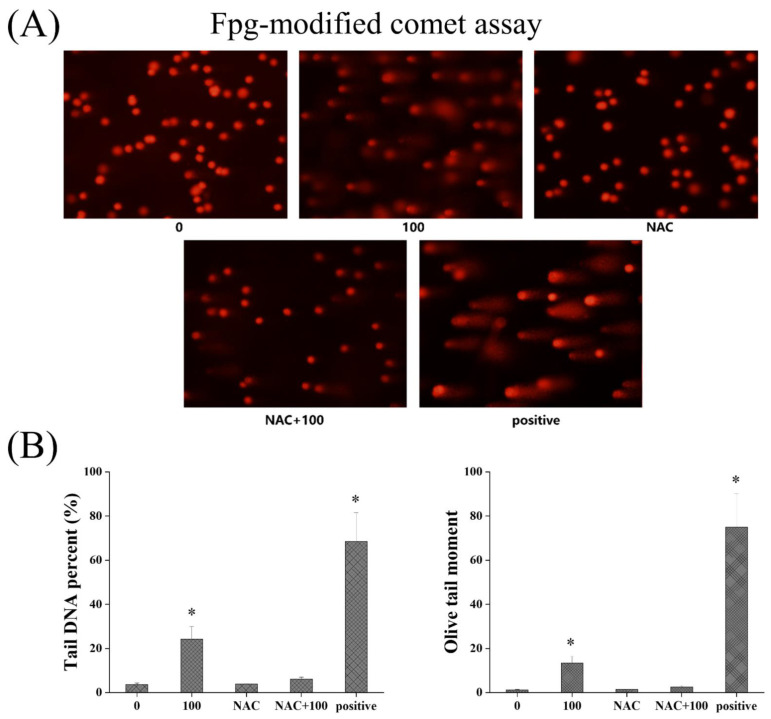
Effects of TiO_2_ NPs on DNA damage in BEAS-2B cells after antioxidant intervention using the Fpg-modified comet assay (**A**). The DNA damage induced by TiO_2_ NPs (100 µg/mL) was significantly recovered after NAC antioxidant intervention. Significant difference from the control (* *p* < 0.05) (**B**).

**Figure 8:**
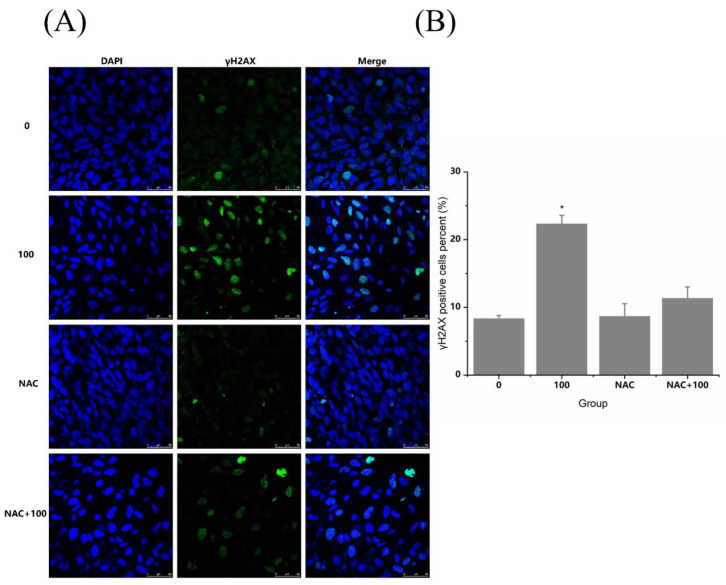
Effects of TiO_2_ NPs on intracellular γ-H_2_AX in BEAS-2B cells after antioxidant intervention. (**A**,**B**) NAC intervention reversed the effect of TiO_2_ NPs on γ-H_2_AX, showing no difference between the TiO_2_ NP+NAC group and the control group on the γ-H_2_AX positive cell rate. Significant difference from the control (* *p* < 0.05).

After antioxidant intervention by NAC, the oxidative stress induced by TiO_2_ NPs was significantly recovered. The coincubation of 100 µg/mL TiO_2_ NPs and 5 mM NAC significantly reduced the level of cellular ROS and restored the significantly reduced ratio of GSH/GSSG. Then, as shown in Figure 7, the results showed that the DNA damage induced by TiO_2_ NPs was also significantly recovered after NAC antioxidant intervention. Using the Fpg-modified comet assay, it was found that no significant difference in the Olive tail moment and comet tail DNA percentage existed between the NAC+TiO_2_ NP coincubation group and the control group, indicating that NAC intervention could alleviate the DNA oxidative damage by TiO_2_ NPs. Meanwhile, as shown in Figure 8, NAC intervention also reversed the effect of TiO_2_ NPs on γ-H_2_AX, showing no difference between the TiO_2_ NP+NAC group and the control group on the γ-H_2_AX positive cell rate. NAC intervention could alleviate the DNA double-strand breaks caused by TiO_2_ NPs. Therefore, oxidative stress should be an important mechanism of TiO_2_ NP-induced cellular genotoxicity.

## 4. Discussion

The present study aimed to investigate the genotoxicity of TiO_2_ NPs, especially through occupational respiratory exposure. In view of previous studies showing that TiO_2_ NP exposure was more likely to induce DNA damage than chromosomal damage [21,28,29], this study focused on DNA damage, especially DNA oxidative damage, to explore more sensitive genotoxicity endpoints and evaluation methods for TiO_2_ NPs. As a result, we found that oxidative stress played an important role in the mechanism of TiO_2_ NP-induced genotoxic effects, and DNA oxidative damage should be a more sensitive genotoxic endpoint for TiO_2_ NP exposure. This was because when the result of the normal comet assay in BEAS-2B cells was negative, the result of the concurrent Fpg-modified comet assay that specifically detected DNA oxidative damage was positive. Moreover, antioxidant intervention could well reverse the DNA damage induced by TiO_2_ NPs. This finding has important implications for nanomaterial genotoxicity studies. Due to the small size and large specific surface area, most nanomaterials can easily induce oxidative stress when interacting with living organisms [7,30,31,32]. Therefore, oxidative stress may be a common pathway for nanomaterials to induce genetic damage and DNA oxidative damage is expected to become a more commonly used genetic endpoint for evaluating the genotoxicity in nanomaterials.

The genotoxicity in nanomaterials has received much attention from the nanotoxicology scientific community [7,8,9,33]. As nanomaterials are different from traditional chemical mutagens, it is very important to find more suitable methods for genotoxicity assessment of nanomaterials [34]. The OECD specially organized experts to discuss the genotoxicity evaluation methods of nanomaterials and finally released some consensus [26], which was fully considered in the design of this study. First, it is necessary to consider the exposure route of nanomaterials and try to choose the route most applicable to human exposure(s). This is because there are insufficient data to recommend one route of administration over another. Therefore, human normal bronchial epithelial cells (BEAS-2B) were selected for this study due to the characteristics of occupational human exposure. As for why the in vitro method was used instead of in vivo experiments, the 3R principle that the use of animals should be minimized was considered and it is necessary to explore some new cell lines for research on the genotoxicity in nanomaterials [35], rather than several fixed traditional cell lines, such as V79 cells. The most suitable cell lines for the evaluation of the genotoxicity in nanomaterials have not yet been determined. In addition, a very important knowledge gap in nanogenotoxicity research is that there could be developed in vitro test methods suitable for detecting secondary genotoxicity [36]. This study showed that in vitro studies could detect the secondary genotoxicity in nanomaterials, especially those mediated by oxidative stress.

Cytotoxicity testing is necessary for determining the top concentration to be applied for in vitro tests of nanomaterials to ensure that genotoxicity is not associated with cytotoxicity [37]. In this study, the CCK8 method was used to detect the cytotoxicity of TiO_2_ NPs and to lay a foundation for the dose selection of subsequent genotoxicity studies. The results showed that the cytotoxicity of TiO_2_ NPs was related to the exposure dose and exposure time, which was consistent with a lot of the published literature [10,38]. The cytotoxicity of TiO_2_ NPs has been demonstrated in various cells, such as human and rat liver cells [39], human lung cells [35], murine fibroblast (LA-9) cells [25], rat bone marrow mesenchymal stem cells (rBMSCs) and rat adipose mesenchymal stem cells (rATSC) [40]. Cytotoxicity was determined by the physicochemical properties, exposure concentration and time of nanoparticles [23,41]. Coarse particles of titanium dioxide or nanoparticles exposed to low doses for long-term exposure have also shown low cytotoxicity [42,43]. The mechanism of cytotoxicity may be related to oxidative stress, inflammatory response and genotoxicity induced by TiO_2_ NPs [29,44]. The cytotoxicity is closely related to its genotoxicity. When cytotoxicity occurs, whether it is apoptosis or necrosis, it will cause an increase in low-molecular-weight DNA fragments in the early stage of cells, thereby increasing the mobility of DNA molecules in electrophoresis, resulting in false-positive results of comets [45]. Therefore, it is not advisable to choose a dose that is too cytotoxic for genotoxicity experiments. According to the international standard (ISO 10993-5), it can be considered that significant cytotoxicity occurs when the cell viability in the treatment group is lower than 70% of the control group [46]. Therefore, in this study, 100 µg/mL was selected as the highest exposure dose and 48 h was selected as the exposure time for subsequent genotoxicity experiments under the condition that the cell viability was >70%.

The extent of cellular uptake is critical for interpreting the genotoxicity results of nanomaterials [37]. Most engineered nanomaterials (ENMs) are insoluble or poorly soluble and are prone to agglomeration in solution systems, so whether they can be taken up by cells and their distribution in cells will determine whether they can directly interact with the genetic material of cells. In general, a lack of uptake in mammalian cells may indicate lower risk of direct genotoxicity or primary genotoxicity. In the present study, nanoparticle characterization showed that the TiO_2_ NPs aggregated into larger particles in DMEM and it may take a long time to enter the cell membrane, so it did not show obvious cytotoxicity before 24 h of exposure. Combined with the results of TEM observation, it was found that the number of nanoparticles entering cells was positively correlated with the exposure dose, which corresponded to the dose-dependent cytotoxicity of TiO_2_ NPs after exposure for 48 h. This explained, to a certain extent, why TiO_2_ NPs needed to be exposed for 48 h to induce a significant decrease in cell activity. The uptake of TiO_2_ NPs by cells could induce an increase in intracellular ROS, which caused a series of negative health effects, and ultimately, led to a decrease in cell viability, resulting in cytotoxicity. Meanwhile, TEM observation also found that TiO_2_ NPs could enter cells, but they did not enter the nucleus. Numerous studies have shown that different types of TiO_2_ NPs can enter the cytoplasm and cause cytotoxicity [29,47,48,49]. However, Hackenberg et al. [50] found that 4% of the particles could enter the nucleus of human nasal mucosal cells without causing DNA breakage. Therefore, it was still suggested that the genotoxicity induced by TiO_2_ NPs in BEAS-2B cells did not primarily act directly with genetic material, while indirect pathways, such as oxidative stress, may be predominant. Since nanoparticles with different crystal sizes and even different types of cells have an impact on the experimental results [51], the possibility of direct interaction between TiO_2_ NPs and DNA cannot be ruled out at present.

Redox balance is critical for maintaining normal cellular physiological functions [52], and moderate ROS levels play a key role in cell signaling, which regulates cell proliferation and survival [53]. GSH reduces hydrogen peroxide and lipid peroxides by donating electrons and is simultaneously oxidized to GSSG. GSSG can obtain electrons from NADPH under the action of GSH reductase and be reduced to GSH. Therefore, the GSH/GSSG ratio is considered to be an important indicator in response to oxidative stress. Oxidative stress is closely related to the cytotoxicity of TiO_2_ NPs. Sha et al. [39] found that TiO_2_ NPs could induce cytotoxicity in different cell lines, including four types of human and rat hepatocytes, which showed good dose–response relationship. Meanwhile, the level of oxidative stress in cells manifested by the increase in ROS and the decrease in GSH was significantly correlated with cytotoxicity, suggesting that oxidative stress may be the main mechanism for the toxic effects of TiO_2_ NPs. The mechanism of ROS generation after TiO_2_ NPs exposure may be related to the disorder of crystal electron configuration at the nanoscale and the easy establishment of electron-donor/acceptor active groups [54]. In vitro studies by Hu et al. [31] showed that oxidative stress induced by TiO_2_ NPs was closely related to endoplasmic reticulum (ER) stress, and ROS production was simultaneously inhibited after 4-phenylbutyric acid was used to inhibit ER stress. Bhattacharya et al. tested the ability of TiO_2_ NPs to generate ROS in non-cellular systems by electron spin resonance (EPR) and found that TiO_2_ NPs only produced a small amount of ROS, suggesting that ROS induced by TiO_2_ NPs should be cell dependent. The present study also found that TiO_2_ NP exposure could induce oxidative stress in BEAS-2B cells, which may be one of the mechanisms for the genotoxic effect in TiO_2_ NPs.

The role of oxidative stress in the genotoxicity of TiO_2_ NPs was further confirmed through antioxidant intervention with NAC. NAC is a commonly used antioxidant that is widely used both experimentally and medically. The antioxidant effect of NAC is manifested in two aspects: its free sulfhydryl group can directly scavenge ROS by interacting with the electrophilic group of ROS and it is also the synthetic precursor of GSH, which can enhance the antioxidant capacity of cells [55]. Xue et al. [56] found that NAC intervention could effectively reduce the oxidative stress caused by TiO_2_ NPs, antagonize their cytotoxicity and protect and reduce apoptosis in vitro. In this study, NAC at a concentration of 5 mM was used for intervention and the results showed that NAC had a good antioxidant capacity and could reduce the genotoxic effect induced by TiO_2_ NPs at the same time. Therefore, the genotoxicity of TiO_2_ NPs may be dominated by oxidative-stress-mediated indirect mode. Meanwhile, antioxidant intervention, such as NAC coincubation, would be an effective way to reduce the genotoxicity in nanomaterials.

The strength of this study was that it revealed that DNA oxidative damage was a sensitive endpoint for the genotoxicity of TiO_2_ NPs through different comet assay methods. The normal comet assay can detect direct DNA single/double-strand breaks, while the endonuclease-modified comet assay can detect specific types of DNA damage. Fpg is a multifunctional DNA base excision repair enzyme that can extensively remove oxidatively damaged bases [57]. Incubating cells with Fpg allows specific excision and fragmentation of oxidatively damaged parts of DNA, which can then be detected using electrophoresis. Moreover, we further demonstrated that oxidative stress played an important role in the mechanism of TiO_2_ NP-induced genotoxic effects through a series of experiments and interventions. This also provided a theoretical basis for the conclusion that DNA oxidative damage was a more sensitive endpoint for genotoxicity of TiO_2_ NPs. The genotoxicity of nanomaterials still lacks standard methods [58]. Based on the full consideration of the consensus of OECD experts, this study proposed a more sensitive genotoxicity endpoint and method for nanomaterials, which would provide some new ideas for the later establishment of standard methods. However, there were still some limitations in this study. It is difficult for in vitro experiments to completely replace in vivo experiments on genotoxicity [59], so it is difficult for this in vitro study to simulate the exposure of human lungs in a real environment. Under the regulation of various systems in vivo, such as macrophage assistance and immune system regulation, most TiO_2_ NPs may be cleared by the body. For in vitro cell experiments with nanomaterials, computational models to calculate more accurate exposure dose metrics have been reported [60,61]. In this study, the step-by-step protocol detailed in the literature [61] was used to determine in vitro dose metrics for TiO_2_ NPs in a 96-well plate, with an exposure time of 48 h (see Tables S1 and S2 in Supplementary Materials for details). The fraction of the particles deposited to reach the cells was estimated to be 0.75 (Figure S1 in Supplementary Materials). Meanwhile, the toxicity of nanoparticles is closely related to their size. If the agglomeration of TiO_2_ NPs in solution is inhibited by dispersants, it is possible to make them enter the nucleus and directly interact with DNA. Therefore, it is still necessary to simulate occupational exposure conditions through animal experiments or conduct epidemiological studies of occupational TiO_2_ NP exposure.

## 5. Conclusions

This study demonstrated that TiO_2_ NPs could induce DNA damage in BEAS-2B cells mediated by oxidative stress and DNA oxidative damage was a more sensitive endpoint for genotoxicity in TiO_2_ NPs. TiO_2_ NPs could be taken up by BEAS-2B cells, but they did not enter the nucleus. Oxidative stress may be the predominant and common indirect pathway for nanomaterials to induce genetic damage. This study provides some new ideas for developing standard methods of nanogenotoxicity research and safety evaluation. 

## Data Availability

The data presented in this study are available on request from the corresponding author.

## Supplementary Materials

The following are available online at https://www.mdpi.com/article/10.3390/nano12152616/s1, Figure S1: Fate and Transport modeling results. (a) Well-bottom TiO_2_ NP concentration over time of simulation. (b) Fraction of TiO_2_ NPs deposited over time of simulation, Table S1: Characterization of TiO_2_ NPs, Table S2: Parameters used in the DG model for computing particle deposition.
