# Supplementary material for: DNA Oxidative Damage as a Sensitive Genetic Endpoint to Detect the Genotoxicity Induced by Titanium Dioxide Nanoparticles
Source: Nanomaterials (Basel). 2022 Jul 29;12(15):2616. doi: 10.3390/nano12152616 (PMC9370504; doi:10.3390/nano12152616)
Supplement: Supplementary file 1 [file nanomaterials-12-02616-s001.zip › nanomaterials-1812087-supplementary.pdf]

nanomaterials

MDPI

Supplementary Materials: DNA Oxidative Damage as a Sensitive Genetic Endpoint to Detect the Genotoxicity Induced by Titanium Dioxide Nanoparticles

Zhangjian Chen, Jiaqi Shi, Yi Zhang, Shuo Han, Jiahe Zhang and Guang Jia *

Determination of in vitro dose metrics for TiO_2 NPs in a 96-well plate with an exposure time of 48 h.

We found detailed research with calculation methods to calculate the in vitro dose metrics [1]. The step-by-step protocol detailed in the literature was used to determine in vitro dose metrics for TiO_2 NPs in a 96-well plate with an exposure time of 48 h. The TiO_2 NPs for this study were characterized in detail. The powder characterization and colloidal characterization in DMEM + 10% (vol/vol) FBS are presented in Table S1.

The effective density (ϱ_{EV}) for the TiO_2 NPs in DMEM + 10% (vol/vol) FBS referred to the value of previous studies [2], which was 1.251.

The effective density- and volume-weighted size distribution determined above were used to determine the dose using the Distorted Grid (DG) model. In the MATLAB software, using a simulator file provided by the literature, the list of sizes and corresponding volume-weighted fractions (from DLS) and effective density were assigned to the relevant variables, and all other variables were set as a default value. Parameters used in the DG model for computing particle deposition are summarized in Table S2. The simulation was then run and data were exported to the excel file.

The fraction deposited and mass concentration vs. time plots automatically generated by the program are shown in Figure S1. From these plots, it is clear that the agglomerates sediment relatively quickly to reach a maximum equilibrium concentration at the bottom of the well (22.67 mg/ml) and fraction deposited (0.75) at ~8 h.

Table S1. Characterization of TiO_2 NPs.

TiO_2 NPs powder properties	Colloidal properties in DMEM + 10% (vol/vol) FBS					
SSA (m^2/g)	d_{BET} (nm)	d_{XRD} (nm)	d_H (nm)	PdI	ζ (mV)	ϱ_{EV}
77.51	18.30	25.12	878.93	0.23	-15.20	1.251

ζ, zeta potential; σ, specific conductance; ϱ_{EV}, effective density; d_{BET}, primary particle diameter determined from SSA; d_H, hydrodynamic diameter measured by DLS; d_{XRD}, particle diameter measured by X-ray diffraction; PdI, polydispersity index; SSA, specific surface area measured by nitrogen adsorption by the Brunauer–Emmet–Teller (BET) method.

Table S2. Parameters used in the DG model for computing particle deposition.

	Parameter	Value	Units
solvent properties	solvent dynamic viscosity	0.00074	Pa s
	density of solvent	1.0	g/cm^3
	temperature of solvent	37.0	°C
particle properties	density of raw material	4.23	g/cm^3
	diameters (dH) of particle/agglomerate species	878.93	nm
	fraction of particle/agglomerate species	1.0	
	agglomerate effective density	1.251	g/cm^3
experimental parameters	height of suspension column	3.0	mm
	initial total concentration of material	0.1	mg/cm^3
	total time of simulation	48	h
	N x g (for centrifugation)	1.0	
model parameters	height of subcompartment (simulation element)	0.005	mm
	time interval for simulation	0.5	s

output data parameters	output data/graph report time interval	30	min
	output compartment height	0.01	mm
	plot/do not plot	1	
	bottom output only	0	
advanced model parameters	sed. Coeff. Concentration dependence	0.0	
	diff. coeff. Concentration dependence	0.0	
	initial dissolution fraction	0.0	
	method for 2odelling dynamic dissolution	1	
	type of dissolution rate	0	
	rate of dissolution	0.048	
	times for dissolution fraction data	[0.0, 12.0]	h
	dissolution fractions corresponding to specified times	[0.0, 0.163 – 0.163]	
	stickiness	0.0	
	adsorption dissociation constant	1.0E-09	

a

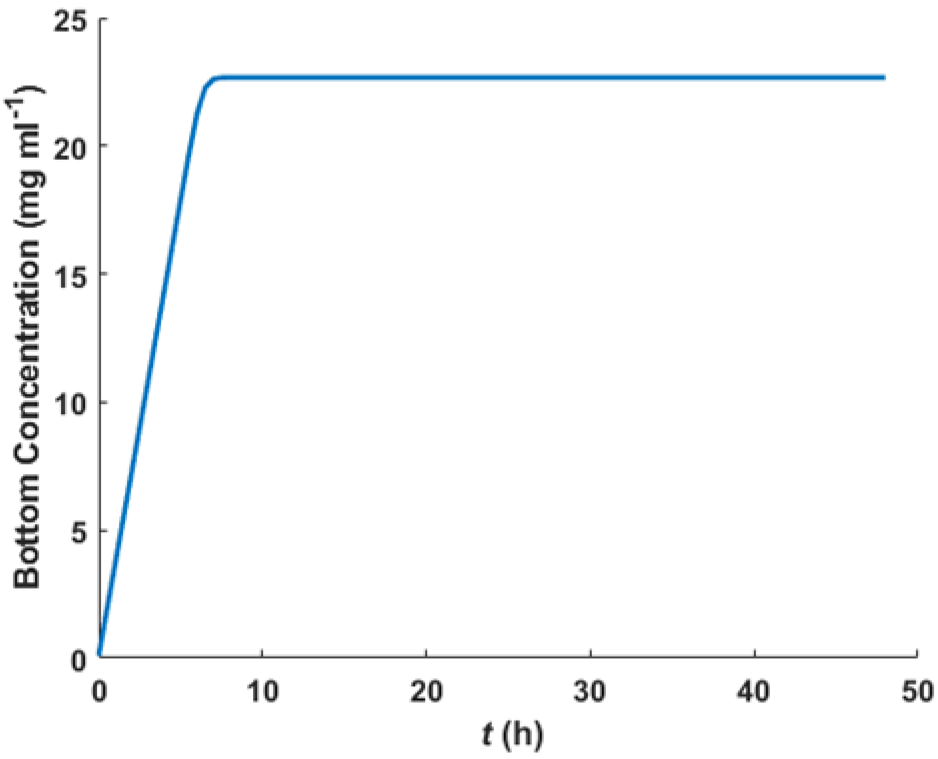

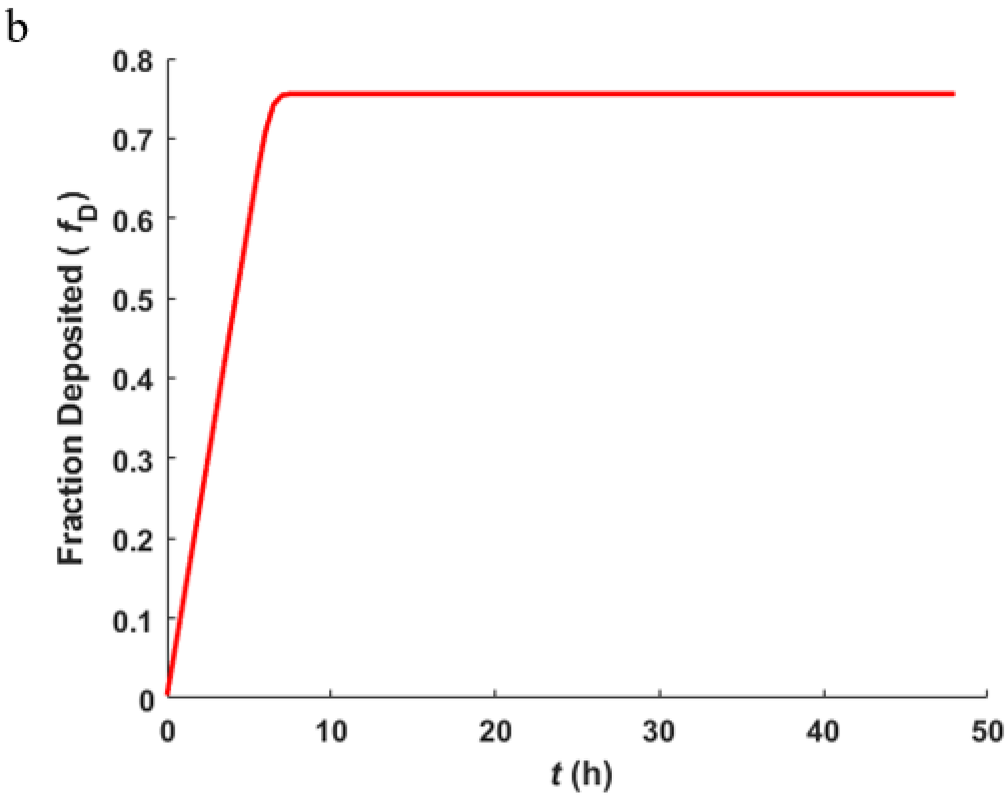

Figure S1. Fate and Transport modeling results. (**a**) Well-bottom TiO_2 NP concentration over time of simulation. (**b**) Fraction of TiO_2 NPs deposited over time of simulation.

Reference

1. DeLoid, G.M.; Cohen, J.M.; Pyrgiotakis, G.; Demokritou, P. Preparation, characterization, and in vitro dosimetry of dispersed, engineered nanomaterials. *Nat. Protoc.* **2017**, *12*, 355–371.
2. DeLoid, G.M.; Cohen, J.M.; Pyrgiotakis, G.; Pirela, S.V.; Pal, A.; Liu, J.; Srebric, J.; Demokritou, P. Advanced computational modeling for in vitro nanomaterial dosimetry. *Part. Fibre Toxicol.* **2015**, *12*, 32.
